# Falsely elevated serum estradiol in woman of reproductive age led to unnecessary intervention and delayed fertility opportunity: a case report and literature review

**DOI:** 10.1186/s12905-022-01828-5

**Published:** 2022-06-16

**Authors:** Jing Zhang, Liangzhi Xu, Lin Qiao

**Affiliations:** 1 Department of Reproductive Endocrinology, West China Second University Hospital, Chengdu, Sichuan People’s Republic of China; 2 Reproductive Endocrinology and Regulation Laboratory, West China Second University Hospital, Chengdu, Sichuan People’s Republic of China; 3 Key Laboratory of Birth Defects and Related Diseases of Women and Children (Sichuan University), Ministry of Education, Chengdu, People’s Republic of China; 4 Department of Obstetrics and Gynecology, West China Second University Hospital, Sichuan University, Sichuan 610041 Chengdu, People’s Republic of China

**Keywords:** Estradiol, Competitive chemiluminescent immunoassay, Immunoassay interference, Heterophile antibody, Case report

## Abstract

**Background:**

The optimal management of patients in reproductive endocrinology relies on the accuracy and validity of sex hormone assays. Endogenous or exogenous substances can compete with the analyte. This competition can result in interfering errors and falsely indicate elevated serum levels. Obvious interference in estradiol assays appears to occur rarely. Consequently, clinicians who are not familiar with the potential of interference could be misled. In addition to unnecessary investigations and interventions and severe mental stress, falsely elevated estradiol results can result in missed or delayed fertility opportunities.

**Case:**

A 28-year-old female with pregnancy demand was diagnosed with polycystic ovary syndrome, Hashimoto’s thyroiditis and subclinical hypothyroidism. She was found to have persistently elevated levels of serum estradiol in the early follicular phase (between 527 and 642 pg/mL). Screening workup was performed for nearly 11 months to find the causes. Serum tumor biomarkers were normal. Abdominal and pelvic computed tomography were negative for adrenal or adnexal masses. A left mesosalpinx cyst and benign pathological results were achieved by laparoscopic surgery. Hormonal substances and dietary supplements were absent, as determined by dietary records. Ultrasound confirmed follicles could grow slowly and eventually ovulate. Falsely elevated estradiol levels were suspected due to the discrepancy among high estradiol levels, follicle growth and normal gonadotropin levels. Immunological interference by heterophile antibody was finally verified by two competitive chemiluminescent immunoassay platforms (estradiol levels in the early follicle phase: 619 pg/mL, Siemens ADVIA CENTAUR and 60 pg/mL, Beckman, DxI 800). Successful clinical pregnancy was eventually achieved by combining induced ovulation, ultrasound monitoring and intercourse guidance.

**Conclusions:**

Analytical interference and laboratory error should be suspicious at first when the clinical characteristics contradict the laboratory results of serum hormones. Measuring serum estradiol with another immunoassay platform is an easy and non-time-consuming method to exclude the heterophile interfering.

## Introduction

The optimal management of patients in reproductive endocrinology relies on the accuracy and validity of sex hormone assays. If the laboratory results contradict the clinical manifestation, clinicians should consider analytic interference and that the laboratory accuracy was not reliable. The prevalence of analytical interference ranges from 0.05 to 6% [1]. Endogenous or exogenous substances can compete with the analyte and result in interfering errors, such as heterophilic antibodies, autoantibodies, rheumatoid factor, bovine alkaline phosphatase, cross-reacting substances [2, 3].

False elevation of serum estradiol due to analytical interference is rare and is most commonly associated with cross-reacting substances, such as the aromatase inhibitor exemestane or the selective estrogen receptor degrader fulvestrant [4, 5]. To date, only nine cases of falsely elevated estradiol due to test interference have been reported previously, and seven of these cases were definitively due to the heterophilic antibody. For woman of reproductive age, in addition to unnecessary investigations and interventions and severe mental stress, falsely elevated estradiol results can even result in missed or delayed fertility opportunities.

## Case

A 28-year-old female visited a local hospital for preconception care in June 2019 because spontaneous abortion previously occurred at the 8th gestational week in February 2019. Hashimoto’s thyroiditis and subclinical hypothyroidism were found, and euthyrox was prescribed (25 µg/day). Sex hormones were also tested on the 22nd day of the cycle for irregular menstruation (cycle ranged from 31 to 51 days), and the results were abnormal with no evidence of dominant follicle or ovulation (Table 1).

**Table 1:** Serum estradiol values as measured with the different assays

Date	Day of cycle	E_2_ (pg/mL)	P (ng/mL)	LH (IU/L)	FSH (IU/L)	Immunoassay method
2019/6/21	22	50	1.18	8.64	7.6	Beckman UniCel DXI
2019/12/1	3	527	0.48	2.9	6.8	Siemens Centaur XP
2019/12/27	2	627	0.61	4.6	6.3	Siemens Centaur XP
2020/1/2	8	574	0.52	7.6	6.8	Siemens Centaur XP
2020/1/29	4	642	0.63	7	7.6	Siemens Centaur XP
2020/3/1	5	579	0.88	5.4	6.9	Siemens Centaur XP
2020/6/25	2	580	0.56	3.5	6.5	Siemens Centaur XP
2020/9/2	3	600	0.72	6.2	7.5	Siemens Centaur XP
2020/12/31	14	619	0.65	8.2	7.9	Siemens Centaur XP
2020/12/31	14	60	0.77	6.86	8.99	Beckman UniCel DXI

E_2_, estradiol; P, progesterone; LH, luteinizing hormone; FSH, follicle stimulating hormone

Then she was referred to the reproductive endocrinology outpatient department of a tertiary teaching hospital for further diagnosis. Menarche occurred at 12 years of age. Physical examination showed a well-developed woman with normal breast size and no signs of hyperandrogenism or insulin resistance. Serum testosterone, androstenedione, and adrenal androgen levels were normal. The pelvic ultrasound on the 4th day of the spontaneous cycle showed a normal uterus size with an endometrium measured at 6 mm. The antral follicle count per ovary was > 20, and a cyst (1.6 × 1.3 × 2.2 cm) beside the left ovary was observed. Serum antimullerian hormone (AMH) was 8.33 ng/mL. Polycystic ovary syndrome was primarily diagnosed based on ovulatory dysfunction and polycystic ovarian morphology after excluding other etiologies. Oral contraceptives containing drospirenone and ethinylestradiol were prescribed for 3 cycles.

After stopping oral contraceptives, the early follicular phase serum estradiol levels persistently increased to between 527 and 642 pg/mL (normal range in follicle phase, 19.5-144.2 pg/mL) during the next 10 months, as illustrated in Table 1. All estradiol measurements were obtained from the same laboratory, using the same competitive chemiluminescent immunoassay (CLIA, Siemens ADVIA CENTAUR). The patient was further referred to the oncology department. However, the levels of other tumor biomarkers were negative (CA-125 6.8, CA-199 6.9, CEA 0.7, β-HCG 2, a-fetoprotein < 1.3). Radiologic assessment by abdominal and pelvic computed tomography was negative for any significant adrenal or adnexal masses, except for a cyst next to the left ovary. Moreover, any form of hormonal substance or dietary supplement was absent by reviewing the dietary diary. Findings on physical examination were completely normal, with no spider angiomas, telangiectasia, palmar erythema, breast tenderness or varicose veins.

On the basis of persistent elevated estradiol and the cystic lesion beside the ovary, a granulosa cell tumor of the ovary was suspected. Laparoscopy surgery was performed, but a left mesosalpinx cyst and benign pathological results were found.

The source of increased estradiol was still unclear after screening for 11 months. The patient was then suggested to monitor the follicle growth by ultrasound and attempt pregnancy. Surprisingly, the follicle would grow slowly and eventually ovulate. An appropriate rise in estradiol was observed with follicle growth (1067.8 pg/mL when the follicle was 1.5 cm in diameter).

There were discrepancies between the serum estradiol level and the clinical picture (follicle growth and normal follicle stimulating hormone). We suspected a case of falsely elevated estradiol levels. The comparison of samples drawn on the same day (14th day of spontaneous cycle), showed our laboratory and the reference laboratory estradiol values to be 619 pg/mL (Siemens ADVIA CENTAUR) and 60 pg/mL (Beckman, DxI 800), respectively. The latter values of estradiol were in concordance with the clinical situation. Thus, falsely increased estradiol was confirmed, and it resulted from analytic interference. Further treatments, including induced ovulation by using letrozole, ultrasound monitoring and intercourse guidance, were scheduled for her urgent pregnancy demand. Successful clinical pregnancy was achieved after six months of therapy. The patient has given birth a healthy baby on 13th March 2022, and she is satisfied with the treatment.

## **Discussion and conclusions**

Although it has been reported in the literature, obvious interference in estradiol assays appears to occur rarely. Consequently, unsuspicious clinicians who are not familiar with the potential of interference could be misled.

We present the case of a 28-year-old female with high estradiol levels inconsistent with the clinical reproductive endocrinological characteristics (unsuppressed follicle growth and gonadotropin values), raising a suspicion of laboratory error. Regrettably, falsely elevated estradiol was not first suspected, although the estradiol level was low before contraceptive therapy. Failure to give proper credence to menstrual and follicle states and to pursue other possible causes for the increased estradiol level resulted in a prolonged period of psychologic stress, unnecessary surgical intervention, and delayed pregnancy attempt for this female of reproductive age.

Analytical interference is a drawback of many hormonal tests, and can result in erroneous values that may lead to unnecessary investigations, misdiagnosis and interventions. The presence of exogenous steroids and metabolites in the circulation is another possible cause for assay-specific interference. However, the patient did not use any other medication; in particular, no estrogen analogues or special food supplements were used.

Heterophile antibodies are endogenous antibodies that can bind reagent immunoglobulins and other components used in immunoassays, and then indiscriminately affect assays. These antibodies may occur naturally without known cause or may result from vaccination, infection, contact with animals, usage of animal immunoglobulins and autoantibodies, such as rheumatoid factor [6]. However, this patient also denied autoimmune disorders, animal exposure or immunoglobulin usage. The patient also showed normal serum sex hormone binding globulin (SHBG) levels, excluding extreme SHBG concentrations as a possible cause for interference. The family history was non-contributory.

Clinical awareness to identify the disparity between laboratory results and clinical presentation, and to order further testing is needed. If heterophile antibodies are suspected, several verification methods are available, such as alternative analytical platforms, treatment with heterophile-blocking agents, polyethylene glycol used to precipitate interfering antibodies, serial dilution examining for nonlinearity, and mass spectrometry. In our case, the falsely elevated estradiol was related to the use of the CLIA Siemens ADVIA CENTAUR® method. Heterophile antibody interference may have been specifically directed towards estradiol reagent immunoglobulins unique to this method. Therefore, another CLIA platform was applied to verify the false assay result and the presence of heterophilic antibodies.

To the best of our knowledge, only nine cases of falsely elevated estradiol due to test interference have been reported, and seven of these cases were definitively due to a heterophile antibody. False hyperestrogenism was reported in four reproductive women receiving IVF treatment. Elevated estradiol measured by enzyme-linked immunosorbent assay was attributed to anti-rabbit IgG heterophil antibody in two females [7]. Heterophilic antibodies of the Elecsys immunoassay from Roche Diagnostics (Mannheim, Germany) were found in the other two females. Of these two, one was from the monoclonal gammopathy [8], and the source of the other was unclear [9]. One perimenopausal (41-year-old) woman with a false high level of estradiol was found to contain an IgA lambda heterophile antibody that could bind the ^125^I-labelled tracer of a competitive radioimmunoassay [10]. One postmenopausal woman with heterophile antibody interference that caused false estradiol elevation was in the setting of a competitive CLIA (Siemens ADVIA CENTAUR®) [11]. In another postmenopausal (62-year-old) woman with multiple steroid hormone elevations, (estradiol, progesterone, testosterone, cortisol) the false estradiol levels were due to laboratory interference of electro-CLIA (Roche Cobas e602) but not by the heterophile antibody [12]. Two adolescents were even reported as having falsely elevated estradiol in a competitive CLIA (Beckman DxI 800) but normal estradiol in the other platforms (Roche e601, Siemens IMULTE, and Abbott ARCHITECT i2000SR) [13].

It is the physician’s responsibility to use laboratory values as an adjunct to the history and physical examination and investigate discrepancies. This case emphasizes the importance of clinical judgement in interpreting unexpected laboratory findings. In addition, maintaining an awareness of heterophilic antibodies and having access to expert laboratory resources will assist in the diagnosis of interference and decrease the potential for unnecessary intervention. Clinicians should contact the laboratory physician for further evaluation of the accuracy of the result before further intervention. Measuring serum estradiol with another immunoassay method is a relatively easy and nontime-consuming method that may reveal immunoassay interference.

We present the case with pregnancy demand, but suffered from the combination of PCOS, Hashimoto’s thyroiditis and subclinical hypothyroidism. It is well known that several clinical indexes are applied to evaluate the reproductive reserve and outcome, such as woman’s age, AMH, follicle stimulating hormone and antral follicle count. However, these parameters are inadequate for PCOS patients because of the oligo-ovulation, even though being with higher AMH or more antral follicles [14]. Therefore, different therapy strategies should be applied to help ovulating and prompting pregnancy, such as ultrasound monitoring and induced ovulation. In addition to the letrozole and clomiphene, other methods or medicines are also proved having positive effect in prompting ovulation, such as life-style adjustment, psychological counseling and insulin sensitizer. Inositol, including D-chiro-inositol and myo-inositol, have been classified as insulin-sensitizers [15]. Moreover, myo-inositol is also essential to produce H2O2 required for the synthesis of thyroid hormones, and may be a suitable therapy for PCOS patients to improve the accompanying endocrine cross-talk disorders, such as insulin resistance and hypothyroidism [16].

Falsely elevated estradiol is a rare but important phenomenon that may lead to unnecessary investigation or intervention or delayed fertility opportunities. Clinicians must have a high index of suspicion when the clinical characteristics contradict laboratory results and must be alert to the potential harm that can occur due to laboratory error.

## Data Availability

All data was included in the published manuscript.

## Abbreviations

AMH: Antimullerian hormone
CLIA: Competitive chemiluminescent immunoassay
SHBG: Sex hormone binding globulin
